# Early Radiographic Progression of Scleroderma: Lung Disease Predicts Long-term Mortality

**DOI:** 10.1016/j.chest.2021.11.033

**Published:** 2021-12-08

**Authors:** Elizabeth R. Volkmann, Donald P. Tashkin, Michael D. Roth, Jonathan Goldin, Grace H.J. Kim

**Affiliations:** a Department of Medicine, David Geffen School of Medicine, University of California, Los Angeles, Los Angeles, CA; b Department of Radiology, David Geffen School of Medicine, University of California, Los Angeles, Los Angeles, CA

**Keywords:** biomarkers, interstitial lung disease, mortality, systemic sclerosis, Dlco, diffusing capacity for carbon monoxide, HR, hazard ratio, HRCT, high-resolution CT, ILD, interstitial lung disease, MMF, mycophenolate mofetil, QILD, quantitative interstitial lung disease, RCT, randomized controlled trial, SLS, Scleroderma Lung Study, SSc, systemic sclerosis

## Abstract

**Background:**

Radiographic end points commonly are included in therapeutic trials for systemic sclerosis (SSc)-interstitial lung disease (ILD); however, the relationship between these outcomes and long-term mortality is unclear.

**Research Question:**

Do short-term changes in radiographic measures of ILD predict long-term survival in patients with SSc?

**Study Design and Methods:**

The Scleroderma Lung Study (SLS) I and II evaluated the safety and efficacy of cyclophosphamide (in SLS I and II) and mycophenolate mofetil (in SLS II) for the treatment of SSc-ILD. Changes in the extent of ILD over time were assessed on high-resolution CT scans of the chest by quantitative image analysis, an approach that applies a computer-based algorithm to assess changes in the radiographic extent of ILD objectively. Participants subsequently were followed for up to 12 years (SLS I) and 8 years (SLS II). Cox proportional hazards models determined whether the change in the quantitative radiographic extent of ILD predicted survival, adjusting for other known predictors of survival.

**Results:**

Among SLS I and II participants, 82 and 90 had follow-up imaging scans, respectively, and were included in the analysis. Participants in both trials who showed an increase in the total quantitative radiographic extent of ILD scores of ≥ 2% at 12 months (SLS I) or 24 months (SLS II) experienced significantly worse long-term survival than those with change scores of < 2% (*P* ≤ .01, log-rank test). In the multivariate Cox models, radiographic progression remained associated with worse long-term survival in SLS I (*P* = .089) and SLS II (*P* = .014).

**Interpretation:**

Data from two independent clinical trial cohorts with extensive long-term follow-up demonstrated that radiographic progression of ILD over 12 to 24 months, in both treatment and placebo arms, can predict increased risk for long-term mortality in patients with SSc. These findings suggest that radiographic end points may serve as surrogates for mortality in SSc-ILD.

## Graphical Abstract

**Figure:**
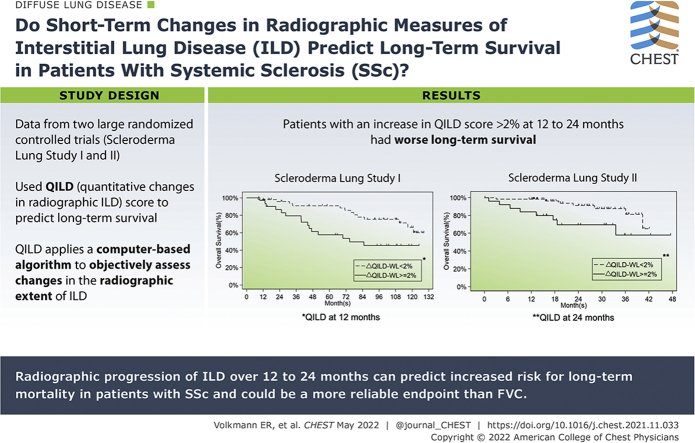

**Take-home Points**

**Study Question:** Do short-term changes in radiographic measures of interstitial lung disease (ILD) predict long-term survival in patients with systemic sclerosis (SSc)?

**Results:** In patients with SSc-ILD enrolled in two large randomized controlled trials, an increase in the quantitative radiographic extent of ILD of ≥ 2% over 1 to 2 years was associated with worse long-term survival.

**Interpretation:** Short-term changes in the radiographic extent of ILD in patients with SSc receiving treatment or placebo may serve as a proxy for long-term mortality. Future SSc-ILD clinical trials should consider including radiographic end points to assess treatment response.

Interstitial lung disease (ILD) is the leading cause of death in systemic sclerosis (SSc)^1^^,^^2^ and a major focus for therapeutic drug discovery in this field. Despite the burgeoning SSc-ILD therapeutic pipeline, no universally accepted end points exist that define an optimal treatment response. End points that directly measure how a patient with SSc-ILD feels and functions are lacking, and although mortality is an unequivocal end point, most SSc clinical trials are powered inadequately and are designed to use mortality as an end point.

The most commonly used surrogate end point in SSc-ILD clinical trials is FVC.^3^, ^4^, ^5^ Although studies have demonstrated that the course of FVC is related to survival in SSc-ILD,^6^^,^^7^ the reliability of this parameter as a direct measure of lung disease may be limited in SSc, where extrapulmonary manifestations can affect its measurement and interpretation substantially (eg, cutaneous sclerosis involving the chest wall, respiratory muscle weakness).^8^ Variations in pulmonary function test protocols, along with patient and technician effort, can influence the reproducibility of the FVC in clinical practice and research further. In addition, studies have demonstrated that the FVC correlates poorly with the actual radiographic extent of ILD in SSc.^9^ Thus, an unmet need exists for the discovery of novel SSc-ILD study end points that are reliable and reproducible and can predict mortality outcomes consistently in patients with SSc.

Objectively quantifying the radiographic extent of ILD may represent a more direct assessment of parenchymal lung disease burden and potentially may obviate the impact of extrapulmonary disease, patient effort, and technical factors that can alter the measurement of FVC, as well as the intrareader and interreader variability in visual radiographic change assessment.^10^ Studies have demonstrated that increased radiographic extent of ILD at baseline predicts responsiveness to immunosuppressive therapy.^11^^,^^12^ Additional studies have found that the radiographic ILD end point is sensitive to change in patients with SSc-ILD undergoing treatment.^13^, ^14^, ^15^, ^16^ However, no studies have evaluated whether a change in the radiographic extent of ILD predicts mortality in this population. To address this issue, the present study examined whether radiographic progression of ILD in patients with SSc receiving treatment predicts long-term mortality. Using data from the Scleroderma Lung Study (SLS) I^3^ and II,^4^ we hypothesized that those patients who experienced increased radiographic progression of ILD over the course of these trials would show worse long-term survival. The findings of this research may broaden our understanding of efficacy assessment in SSc-ILD research and ultimately may shape the design of future clinical trials for this often-fatal disease.

## Study Design and Methods

### Study Participants

All participants enrolled in SLS I^3^ (ClinicalTrials.gov Identifiers: NCT01762449 and NCT00004563) and SLS II^4^ (ClinicalTrials.gov Identifier: NCT00883129) who underwent a follow-up, standardized, high-resolution CT (HRCT) scan of the chest were eligible to participate in this study. SLS I and II were randomized controlled trials (RCTs) that included an ethnically diverse population of both male and female patients with SSc-ILD followed up at multiple centers across the United States. Eligibility criteria for these trials were similar.^3^^,^^4^ The institutional review board of each site approved the primary studies and long-term follow-up. Informed consent was obtained from all participants.

### SLS I and II Study Design

In SLS I, 158 participants were randomized to receive oral cyclophosphamide or placebo for 12 months and were followed up for an additional 12 months off of therapy.^3^ In SLS II, 142 patients were randomized to receive mycophenolate mofetil (MMF) for 24 months or oral cyclophosphamide for 12 months followed by an additional 12 months of placebo.^4^

### SLS I and II Assessment Measurements

The FVC (primary SLS I and II end point) was measured every 3 months during the 24-month study periods.^3^^,^^4^ HRCT thoracic imaging was obtained at baseline and at the conclusion of active treatment in both trials (ie, at 12 months in SLS I and at 24 months in SLS II). A Computer-Aided Design scoring system^13^, ^14^, ^15^ was used to calculate the quantitative ILD (QILD) score for the entire lung at baseline and follow-up (e-Fig 1 shows further details on the HRCT imaging protocol and scoring). The QILD score included the sum of all abnormally classified scores, including fibrosis (eg, reticular opacity with architectural distortion), ground-glass opacity (eg, increased parenchymal attenuation), and honeycombing (eg, clustered air-filled cysts with dense walls).

The QILD score threshold to define radiographic ILD progression (QILD score of ≥ 2%) was determined first by performing a statistical analysis of the cohorts using a quantitative imaging biomarker algorithm proposed in our prior publication.^17^ This analysis examines both technical reproducibility and clinical reproducibility.^17^ Technical reproducibility was reported as 0.60% in the limited agreement (e-Fig 2). Subsequently, the factor of 2.77 (= 1.96 × $\sqrt{2}$) was multiplied by the variability of the two HRCT scans, resulting in a threshold of 1.66%. This number then was rounded up to 2%.

Next, we considered clinically meaningful correlates of a ≥ 2% increase in QILD, such as change in FVC percent predicted, patient-reported outcomes, and mortality. In the SLS I and II cohorts, an increase of QILD score of ≥ 2% was associated with a clinically meaningful decline in FVC percent predicted.^18^ Specifically, most patients meeting the minimal clinically important difference estimates for FVC percent predicted worsening also experienced an increase in QILD score of > 2%. The minimal clinically important difference criteria for FVC worsening (–3.0% to –3.3%) were derived based on an analysis of two valid patient-reported outcomes.^18^ As a final step, we tested the relationship between additional thresholds of QILD score worsening (eg, ≥ 1%, ≥ 3%, ≥ 4%, and ≥ 5%) and survival, and the ≥ 2% increase in QILD score demonstrated the strongest association with survival in both of the SLS I and II cohorts.

### Long-term Mortality

During the SLS I and II trials, mortality data were collected and causes of death were adjudicated by data safety and monitoring boards. After the 24-month trials, patients or their designated surrogates were contacted annually to assess morbidity and mortality outcomes. If the patient or previously designated contact person could not be reached, investigators contacted site investigators and searched publicly available death registries (eg, National Death Index and Social Security Death Index), as well as online obituaries. Survival status was ascertained for up to 12 and 8 years after the commencement of SLS I and II, respectively.

### Statistical Analysis

All tests were 2-sided and were performed using SAS version 9.4 software (SAS Institute).

#### Baseline Characteristics

Summary statistics were generated for baseline characteristics from the two cohorts. Group comparisons were performed using two-sample *t* tests and χ ^2^ tests.

#### Primary Outcome

Survival: The primary outcome was all-cause mortality. The Kaplan-Meier estimate was used to generate survival curves, and the log-rank test was used to compare survival between participants who experienced progression of ILD (≥ 2% increase in QILD score) vs those who experienced stability or improvement of ILD (< 2% increase in QILD score). Cox proportional hazard models subsequently were developed. Using a comprehensive variable selection process described in our previous publication,^7^ the following variables were found to be associated significantly with mortality in univariate analyses in both the SLS I and II cohorts: age, modified Rodnan skin score, and baseline FVC percent predicted. Therefore, the aforementioned variables were included in the final Cox models. Treatment arm assignment was not associated significantly with mortality in SLS I or II^7^; however, given the potential interaction between treatment arm assignment and change in QILD, we created exploratory Cox models that also included treatment arm assignment. We also created exploratory Cox models that included variables associated with mortality based on expert knowledge (eg, sex, race, diffuse SSc subtype, and diffusing capacity for carbon monoxide [Dlco] % predicted).

#### Secondary Outcome

Course of FVC: Mixed-effects models were created to compare the course of the FVC percent predicted (measured every 3 months over 24 months) between patients who experienced progression of ILD (≥ 2% increase in QILD score) vs those who experienced stability or improvement of ILD (< 2% increase in QILD score). Covariates included the baseline FVC percent predicted, treatment arm assignment, and the change in QILD score.

## Results

### Participant Characteristics

Among all of the SLS I (n = 158) and II (N = 142) participants, 82 and 90, respectively, underwent follow-up HRCT scans of the chest and were included in the present analysis. In SLS I, the follow-up HRCT scan was part of a substudy that was delayed in onset, limiting the ability to enroll patients who already had completed the 12-month follow-up visit. In SLS II, the primary reason not all patients had undergone an HRCT scan of the chest at 24 months was participant drop out, death, or both.

The baseline disease features and demographic characteristics of the SLS I and II participants who underwent follow-up HRCT scans were similar and closely reflected those of the overall study population (e-Table 1). Most patients were women with relatively early SSc (approximately 3 years from the onset of the first non-Raynaud symptom attributable to SSc) and a moderate degree of restriction on pulmonary function testing results.

### Radiographic ILD Progression in SLS I and II

In SLS I, 34 participants (41%) experienced an increase in QILD score of ≥ 2% for the entire lung at 12 months. Among these participants, 13 were randomized to cyclophosphamide and 21 were randomized to placebo. This difference between treatment arms is consistent with the statistically significant impact of cyclophosphamide on the change in QILD score at 12 months reported in our prior studies.^12^ In SLS II, 28 participants (31%) experienced an increase in QILD score of ≥ 2% for the entire lung at 24 months. Among these participants, 15 were randomized to cyclophosphamide and 13 were randomized to MMF. In contrast to the outcome in SLS I, in which half of the participants received placebo, no significant difference in treatment effect was observed between the two active treatment arms (MMF and cyclophosphamide) in SLS II.^14^ No significant differences were found in the baseline characteristics of participants who experienced an increase in QILD score of ≥ 2% and those who did not in either SLS I or II, with the exception of a lower baseline Dlco % predicted in SLS I participants who experienced an increase in QILD score of ≥ 2% (Table 1).

**Table 1:** Baseline Patient Characteristics of SLS I and II Participants Who Showed a Change in QILD Score of ≥ 2% vs < 2% at 12 and 24 Months, Respectively

Variable	SLS I			SLS II		
	Change in QILD Score of ≥ 2% (n = 34)	Change in QILD Score of < 2% (n = 48)	*P* Value	Change in QILD Score of ≥ 2% (n = 25)	Change in QILD Score of < 2% (n = 65)	*P* Value
Age, y	48.1 ± 11.2	45.6 ± 11.7	.27	51.63 (9.74)	51.36 (8.98)	.81
Female sex	21 (61.76)	39(81.25)	.050	15 (60.00)	51 (78.46)	.053
SSc duration, mean (SD), y^a^	2.18 (2.42)	3.10 (3.47)	.031	2.50 (2.83)	1.67 (3.00)	.59
Diffuse cutaneous disease	21 (61.76)	27 (56.25)	.62	15 (60.00)	38 (58.46)	.96
Race^b^						
White	21 (61.76)	34 (70.83)	.62	15 (60.00)	44 (67.69)	.32
Black	6 (17.65)	5 (10.42)	...	8 (32.00)	14 (21.54)	...
Asian	2 (5.88)	1 (2.08)	...	1 (4.00)	5 (7.69)	...
Other	5 (14.71)	7 (14.58)	...	1 (4.00)	2 (3.08)	...
Unknown	0 (0.00)	1 (2.08)	...	0 (0.00)	0 (0.00)	...
mRSS	16.53 ± 11.63	14.15 ± 10.78	.33	12.93 ± 7.92	14.20 ± 10.05	.82
History of prior smoking	11 (33.33)	17 (35.42)	.85	6 (25.00)	19 (29.23)	.69
FVC percent predicted	66.67 ± 10.85	71.16 ± 11.68	.21	67.69 ± 7.78	65.91 ± 9.00	.93
Dlco percent predicted^b^	43.20 ± 12.70	50.46 ± 14.54	.022	55.94 ± 13.91	55.42 ± 12.74	.87
QLF % whole lung	8.23 ± 5.98	10.14 ± 10.78	.35	7.77 ± 5.72	7.80 ± 7.01	.97
QILD score % whole lung	31.57 ± 11.99	35.62 ± 17.04	.24	26.61 ± 8.84	27.05 ± 13.91	.86

Data are presented as No. (%), mean ± SD, or median (interquartile range), unless otherwise indicated. Dlco = diffusing capacity for carbon monoxide; mRSS = modified Rodnan skin score; QILD = quantitative interstitial lung disease; QLF = quantitative lung fibrosis; SLS = Scleroderma Lung Study; SSc = systemic sclerosis.

a For disease duration, n = 81 and n = 84 in SLS I and II, respectively.

b For race, n = 81 in SLS I.

**Figure 1:**
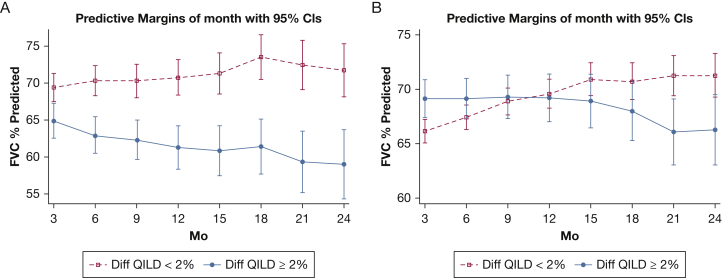
A, B, Graphs showing the mixed-effects model of the course of FVC percent predicted over 24 months for participants with ≥ 2% (red line) vs < 2% (blue line) increase in QILD score for SLS I (A) and SLS II (B). Covariates for both models included the baseline FVC percent predicted and treatment arm. QILD = quantitative interstitial lung disease; SLS = Scleroderma Lung Study.

SLS I participants who experienced an increase in QILD score of ≥ 2% over 12 months also were more likely to experience a decline in FVC percent predicted over the course of the trial, whereas SLS I participants who experienced a change in QILD score of < 2% were more likely to experience stability or improvement in FVC percent predicted (Fig 1A). Similarly, in SLS II, those participants who experienced an increase in QILD score of ≥ 2% over 24 months also were more likely to experience a decline in FVC percent predicted over the course of the trial, whereas those participants who experienced a change in QILD score of < 2% were more likely to experience stability or improvement in FVC percent predicted (Fig 1B).

### Long-term Survival in SLS I and II

In SLS I, 66 patients (42%) of the entire cohort had died within 12 years after the first patient was randomized (cyclophosphamide group, n = 38; placebo group, n = 28). Most deaths (65%) were the result of underlying SSc, and among the deaths resulting from SSc, 67% were the result of respiratory failure. In the SLS I participants with follow-up HRCT scans at 12 months, 28 deaths (34%) occurred during the long-term follow-up period (cyclophosphamide group, n = 15; placebo group, n = 13).

In SLS II, 30 patients (21%) of the entire cohort had died within 8 years after the first patient was randomized (cyclophosphamide group, n = 16; MMF group, n = 14). Most deaths (58%) were the result of underlying SSc, and among the deaths resulting from SSc, 50% were the result of respiratory failure. In the SLS II participants with follow-up HRCT scans at 24 months, 15 deaths (17%) occurred during the long-term follow-up period (cyclophosphamide group, n = 6; MMF group, n = 9).

### Radiographic Progression of ILD Predicts Survival

**Figure 2:**
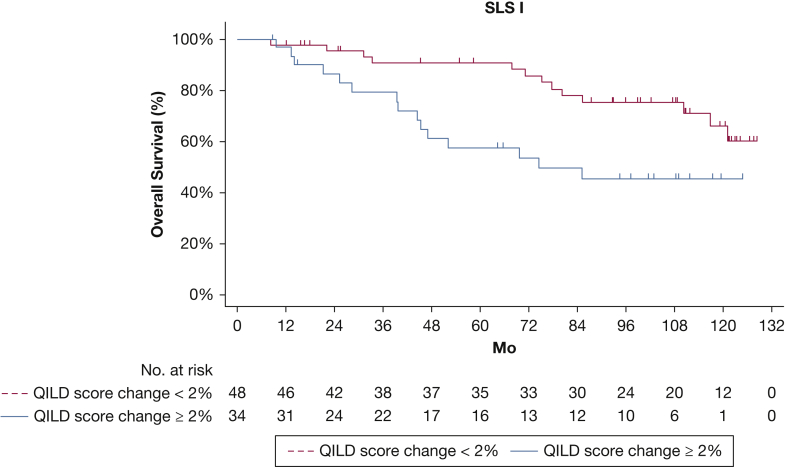
Kaplan-Meier plot showing overall survival for participants with ≥ 2% vs < 2% increase in QILD score for the entire lung from baseline to 12 months in SLS I. QILD = quantitative interstitial lung disease; SLS = Scleroderma Lung Study.

**Figure 3:**
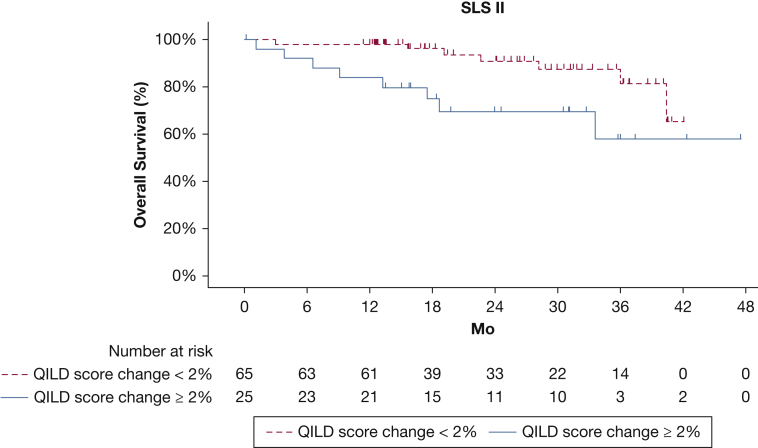
Kaplan-Meier plot showing overall survival for participants with ≥ 2% vs < 2% increase in QILD score for the entire lung from baseline to 24 months in SLS II. QILD = quantitative interstitial lung disease; SLS = Scleroderma Lung Study.

During the 12-year long-term follow-up period, SLS I participants who experienced an increase in QILD score of ≥ 2% for the entire lung at 1 year showed a significantly increased risk of death (*P* = .01, log-rank test) (Fig 2). During the 8-year long-term follow-up period, SLS II participants who experienced an increase in QILD score of ≥ 2% for the entire lung at 2 years showed a significantly increased risk of death (*P* = 0.019, log-rank test) (Fig 3).

After adjusting for age, modified Rodnan skin score, and baseline FVC percent predicted, a suggestive association was found between increase in QILD score of ≥ 2% and mortality in SLS I participants (hazard ratio [HR], 1.98; *P = .*089) (Table 2). In addition, after adjusting for the aforementioned variables, an increase in QILD score of ≥ 2% was associated with a significantly increased risk of mortality in SLS II participants (HR, 3.86; *P = .*014) (Table 3). After adding treatment arm assignment (e-Tables 2, 3), as well as the variables of sex, race, diffuse SSc subtype, and Dlco percent predicted (e-Tables 4, 5) to the aforementioned Cox models, an increase in QILD score of ≥ 2% remained associated with mortality in both SLS I (suggestive association) and SLS II (significant association).

**Table 2:** Cox Proportional Hazards Model for Survival in SLS I (N = 82)

Variable	Hazard Ratio	95% CI	*P* Value	C Index
Univariate analyses				
QILD score change of ≥ 2%	2.61	1.23-5.57	.013	0.637
Continuous change in QILD score	1.02	0.99-1.06	.16	0.643
Multivariate analyses				
Age	1.04	1.01-1.08	.019	0.728
mRSS	1.04	1.00-1.07	.028	
Baseline FVC percent predicted	0.97	0.93-1.01	.098	
QILD score change of ≥ 2%	1.98	0.90-4.40	.089	

mRSS = modified Rodnan skin score; QILD = quantitative interstitial lung disease; SLS = Scleroderma Lung Study.

**Table 3:** Cox Proportional Hazards Model for Survival in SLS II (N = 90)^a^

Variable	Hazard Ratio	95% CI	*P* Value	C Index
Univariate analyses				
QILD score change of ≥ 2%	3.17	1.15-8.76	.026	0.688
Continuous change in QILD score	1.10	1.03-1.17	.004	0.735
Multivariate analyses				
Age	1.05	1.00-1.11	.074	0.743
mRSS	1.00	0.94-1.07	.614	
Baseline FVC % predicted	1.01	0.95-1.06	.895	
QILD score change of ≥ 2%	3.86	1.31-11.27	.014	

mRSS = modified Rodnan skin score; QILD = quantitative interstitial lung disease; SLS = Scleroderma Lung Study.

In an exploratory analysis, physiologic progression of ILD was substituted for the baseline FVC percent predicted as a covariate in the Cox model. Physiologic progression was defined according to the Outcome Measures in Rheumatology (OMERACT)criteria as FVC percent predicted decline of ≥ 10% or FVC percent predicted decline of between 5% and 9% and Dlco percent predicted decline of ≥ 15%.^6^^,^^19^ In SLS I, 15 patients met the OMERACT criteria for physiologic ILD progression at 12 months. After adjusting for age, modified Rodnan skin score, and physiologic progression of ILD at 12 months, an increase in QILD score of ≥ 2% remained associated significantly with an increased risk of mortality (HR, 2.28; *P = .*037) (e-Table 6). In another exploratory analysis, we included the change in FVC percent predicted at 12 months (measured continuously) as a covariate, and this covariate was not associated significantly with mortality (*P = .*97) (e-Table 7).

In SLS II, too few patients (n = 3) met the OMERACT criteria for physiologic ILD progression at 24 months; therefore, a Cox model that included this covariate could not be created. However, similar to SLS I, we also created a Cox model that included the change in FVC percent predicted at 24 months (measured continuously) as a covariate, and a suggestive association was found between change in FVC percent predicted with mortality (HR, 0.93; *P = .*06) (e-Table 8).

## Discussion

Using data from two independent cohorts with extensive clinical characterization and follow-up, the present study demonstrated that radiographic progression of SSc-ILD (defined by a ≥ 2% increase in QILD score) over the course of 1 to 2 years is associated with an increased risk of long-term mortality. Even after adjusting for other factors known to affect survival in this patient population, worsening quantitative radiographic extent of ILD remained associated significantly with mortality in SLS II (suggestive association for SLS I).

Individualization of end points in RCTs for SSc-ILD is an evolving area of research with considerable implications for future drug discovery and development. Because no valid patient-reported outcomes currently exist for this disease state and an insufficient number of deaths occur over standard trial periods,^3^, ^4^, ^5^ an unmet need exists for establishing clinically meaningful and reliable surrogate end points for SSc-ILD RCTs.

The FVC has served as the primary end point for three of the largest SSc-ILD RCTs^3^, ^4^, ^5^; however, these trials all used different statistical methods for evaluating FVC change over time. For instance, in SLS I, a generalized estimating equation regression model was created that used imputation for missing data to compare outcomes in the FVC percent predicted over 1 year.^3^ In SLS II, a joint model that combined a mixed-effects model with a survival model to adjust for nonignorable missing data resulting from dropouts, treatment failures, and deaths was used to compare the course of the FVC percent predicted over 2 years.^4^ In the Safety and Efficacy of Nintedanib in Systemic Sclerosis (SENSCIS) trial comparing nintedanib with placebo for SSc-ILD,^5^ the annual rate of decline in FVC (milliliters per year) over 1 year was assessed with a random-coefficient regression model that also used imputation for missing data. In the recently published phase 3 trial of tocilizumab for the treatment of diffuse cutaneous SSc, FVC was a key secondary end point, and that study evaluated the difference in distribution of change from baseline to week 48 in the FVC percent predicted.^16^ The diverse methodologies used to evaluate how various treatments affect lung function render it difficult to compare treatment effects across studies. In addition, studies have demonstrated considerable fluctuations in declines in the FVC percent predicted within individual patients receiving treatment for ILD.^20^ Finally, lung function outcomes correlated poorly with various patient-reported outcomes in some trials,^5^^,^^21^^,^^22^ raising the question of whether lung function change truly is a clinically meaningful end point from the perspective of the patient.

The present study suggests that changes in the quantitative radiographic extent of ILD may serve as a proxy for long-term mortality in patients with SSc-ILD. Moreover, increased radiographic progression of ILD remained associated significantly with increased mortality in the multivariate analysis in SLS II, whereas physiologic progression on pulmonary function testing was not. These findings are consistent with studies of patients with other ILDs.^22^ For example, a recent study demonstrated that longitudinal changes in semiquantitative visual HRCT scan fibrosis scores of > 7% predicted lung transplantation-free survival in patients with idiopathic pulmonary fibrosis followed up for an average of 3 years.^23^ Moreover, progression of ILD abnormalities in participants of the Framingham Heart Study based on visual assessment was associated with increased mortality.^24^ However, the present study is the first study to demonstrate that increased quantitative radiographic progression of ILD beyond a prespecified threshold over the course of both 1- and 2 years was associated with an increased risk of death in patients with SSc.

In addition to predicting survival, a prior study from SLS II demonstrated that changes in the quantitative radiographic extent of ILD in the entire lung were associated significantly with patient-reported outcomes, such as dyspnea.^22^ Specifically, increased QILD score over 2 years was associated with worse scores on the transitional dyspnea index.^22^ Thus, radiographic progression of ILD may closely reflect actual changes in how a patient feels and functions.

In considering the results of the multivariate analyses, the HR for change in QILD score was higher for the SLS II survival model compared with the SLS I model. This finding could suggest that radiographic progression measured at 2 years is a better predictor of long-term mortality than that at 1 year, although to confirm this hypothesis, one would need to measure QILD score changes at these two time points in the same cohort. It is also notable that although substantially more patients randomized to placebo experienced radiographic progression compared with cyclophosphamide, the long-term mortality rates were similar between the SLS I study treatment arms, suggesting that 1 year of therapy is unlikely to lead to a sustained improvement in long-term survival.

Interestingly, the baseline characteristics of study participants who experienced radiographic progression of ILD and those who did not were fairly similar. Although not statistically significant, a greater proportion of men and Black participants experienced radiographic progression of ILD during these studies. These analyses may have been underpowered to detect significant differences, and larger studies are needed to determine whether sex and race affect radiographic progression of SSc-ILD.

Although quantitative imaging analysis can detect changes in the radiographic extent of ILD more sensitively than visual assessment,^25^ a major limitation of this study is that automated methods for radiographic ILD scoring are not yet available widely in clinical practice. However, this approach has been used in a number of SSc RCTs,^3^^,^^4^^,^^16^ demonstrating its feasibility as a study end point. Furthermore, insufficient inspiration, respiratory muscle strain, or both may curtail lung expansion during HRCT scan assessment. Furthermore, superimposed infection also may confound quantitative image assessment. Reassuringly, study technicians were trained to ensure adequate inspiration, and all images underwent quality control assessment to evaluate for the presence of infection before quantitative image analysis. Another potential shortcoming of this study is that radiographic assessment was performed at different time points in SLS I (1 year) and SLS II (2 years), which could introduce lead time bias. However, few deaths occurred during the active treatment periods (n = 5 and n = 16 for SLS I and II, respectively). Moreover, this also could be perceived as a strength because the findings suggest that radiographic changes at either of these time points have prognostic value.

Additional strengths of this study include the evaluation of two multicenter SSc-ILD cohorts who received standard treatment and follow-up during the radiographic assessment period. Moreover, although not all participants underwent a follow-up HRCT scan assessment, more than half of all patients did, and no difference was found in the baseline characteristics of participants without follow-up HRCT scan assessment and the entire study cohort. Finally, to our knowledge, this is the longest period during which participants in any RCT for SSc-ILD have been followed up for mortality outcomes.

## Interpretation

In summary, measuring changes in the quantitative radiographic extent of ILD predicts long-term mortality in patients with SSc. Moreover, radiographic progression of ILD seemed to be a stronger predictor of mortality than longitudinal functional decline in two independent, multicenter cohorts. Radiographic end points may serve as more reliable and reproducible end points in SSc-ILD trials compared with FVC, and this is the first study to suggest that these end points are surrogates for mortality. Future studies are needed to determine whether measuring the change in QILD score at earlier time points (eg, 6 months) could be used to detect treatment effects and predict long-term outcomes in these patients.

## Supplementary Data

e-Online Data
