# Supplementary material for: Early Radiographic Progression of Scleroderma: Lung Disease Predicts Long-term Mortality
Source: Chest. 2021 Dec 8;161(5):1310–9. doi: 10.1016/j.chest.2021.11.033 (PMC9131045; doi:10.1016/j.chest.2021.11.033)
Supplement: e-Online Data [file mmc1.pdf]

CHEST Online Supplement

Early Radiographic Progression of Scleroderma

Lung Disease Predicts Long-term Mortality

Elizabeth R. Volkmann, MD; Donald P. Tashkin, MD; Michael D. Roth, MD; Jonathan Goldin, MD, PhD; and Grace H.J. Kim, PhD

CHEST 2022; 161(5):1310-1319

Online supplements are not copyedited prior to posting and the author(s) take full responsibility for the accuracy of all data.

© 2022 AMERICAN COLLEGE OF CHEST PHYSICIANS. Reproduction of this article is prohibited without written permission from the American College of Chest Physicians. See online for more details. **DOI:** 10.1016/j.chest.2021.11.033

e-Appendix 1.

Supplementary Methods

HRCT Imaging Protocol

A standardized protocol was developed and monitored by the Radiology Core at UCLA in both SLS I and II. In SLS I, non-volumetric HRCT at suspended full inspiration was performed in the prone position. Technologists were trained to coach maximal inspiratory breath-hold from the patients and instructed them to "Take your biggest breath in until you feel your lungs are completely full, in the same way you do in the lung function laboratory, and then signal when you feel completely full and hold your breath." HRCT images were acquired from 5 manufacturers with at least 4 or more multi-detector rows. Exposure directly from HRCT assessment ranged from 80 to 380 mAs (mean of 245 mAs±79) at 120 to 140 kVp. Non-volumetric CT of 1- to 2-mm slice thickness were acquired at 10-mm increments and were typically reconstructed with sharp or over-enhancing filters. In SLS II, volumetric HRCT at suspended full inspiration (as described above) was performed in the prone position with CT parameters of standardized thin-section (<1.25 mm) reduced-dose (80–100 mAs) at 120 kVp. CT images were acquired using 12 different multi-detector CT scanner models from two manufacturers. CT images were under strict quality control guidelines, including credentialing the scanners, by showing stability of water and air measures on two phantom scans and stability of phantom measures on phantoms scanned within 24 hours of each subject's scan.

QILD score is derived in automated pipeline from machine learning. The steps are (1) lung and lobar segmentation, (2) desnoise as part of normalization of multi-center, multi-scanner models, (3) grid-sample of voxel, (4) calculation of selected voxel-wise radiomic (texture features), (5) classification by support vector machine, (6) express a ratio of the number of classified voxels of ILD to the total grid-sample voxels (Supplementary e-Figure 1).

Online supplements are not copyedited prior to posting and the author(s) take full responsibility for the accuracy of all data.

e-Table 1. Patient characteristics of all SLS I and II participants and those who had follow up HRCT scans of the chest and were included in this analysis[a]

	All SLS I (N=158)	SLS I with HRCT (N=82)	All SLS II (N=142)	SLS II with HRCT (N=90)
Age (years)- Mean (SD)	47.5 (12.4)	46.6 (11.5)	52.6 (9.8)	51.4 (9.1)
Female- %	64.6%	73.2%	76.1%	73.3%
SSc Duration (years)- Median (IQR)	2.6 (1.5, 4.7)	2.5 (1.3, 4.5)	1.70 (1.1, 3.8)	2.1 (1.1, 4.0)
Diffuse- N(%)	94 (59.5%)	48 (58.5%)	83 (56.85)	53 (58.9%)
Race- N(%)				
White	105 (66.88%)	55 (67.07%)	99 (67.07%)	59 (65.56%)
AA	26 (16.56%)	11 (13.41%)	32 (22.54%)	22 (24.44%)
Asian	7 (4.46%)	3 (3.66%)	9 (6.34%)	6 (6.67%)
Other	19 (12.10%)	12 (14.63%)	2 (1.41%)	3 (3.33%)
Unknown		1 (1.22%)		
MRSS- Mean (SD)	14.0 (10.9)	15.1 (11.1)	14.4 (10.4)	14.1 (9.6)
FVC % Predicted- Mean (SD)	71.7 (12.1)	70.3 (11.5)	67.0 (9.3)	66.4 (8.7)
DLCO % Predicted- Mean (SD)	47.4 (14.0)	47.4 (14.2)	55.0 (13.1)	55.6 (13.0)
QLF % Whole Lung- Mean (SD)[b]	10.1 (10.2)	9.3 (9.1)	8.60 (6.93)	7.8 (6.6)
QILD % Whole Lung- Mean (SD)[b]	35.2 (16.8)	33.9 (15.2)	29.46 (13.99)	26.9 (12.7)

[a] No significant differences were detected between patients with and without follow-up HRCT scans in SLS I or SLS II.

[b] N=127 and N=137 for all SLS I and II, respectively.

Online supplements are not copyedited prior to posting and the author(s) take full responsibility for the accuracy of all data.

e-Table 2. Cox Proportional Hazards model for survival in SLS I (N=82) with the addition of treatment arm assignment as a covariate[a]

	Hazard ratio	95% CI	P-value
Age	1.04	[1.01, 1.08]	0.021
mRSS	1.04	[1.00, 1.07]	0.030
Baseline FVC%-predicted	0.97	[0.93, 1.01]	0.099
ΔQILD ≥2%	1.98	[0.88, 4.43]	0.097
Treatment arm (CYC)	0.98	[0.44, 2.19]	0.961

[a] C-index for model: 0.728

e-Table 3. Cox Proportional Hazards model for survival in SLS II (N=90) with the addition of treatment arm assignment as a covariate[a]

	Hazard ratio	95% CI	P-value
Age	1.05	[0.99, 1.11]	0.11
mRSS	1.01	[0.95, 1.07]	0.74
Baseline FVC%-predicted	1.00	[0.94, 1.06]	0.87
ΔQILD ≥2%	3.96	[1.35, 11.64]	0.012
Treatment arm, MMF	1.40	[0.45 4.38]	0.563

[a] C-index for model: 0.774

e-Table 4. Cox Proportional Hazards model for survival in SLS I (N=82) including additional covariates[a]

	Hazard ratio	95% CI	P-value
Age	1.05	(1.01, 1.10)	0.015
Male	1.21	(0.52, 2.80)	0.664
African American	1.78	(0.46, 6.95)	0.405
Diffuse	1.06	(0.33, 3.34)	0.925
mRSS	1.03	(0.98, 1.08)	0.229
Baseline DLCO%-predicted	1.02	(0.98, 1.06)	0.263
Baseline FVC%-predicted	0.95	(0.91, 1.00)	0.053
ΔQILD ≥2%	1.99	(0.87, 4.54)	0.102

[a] C-index for model: 0.743

Online supplements are not copyedited prior to posting and the author(s) take full responsibility for the accuracy of all data.

e-Table 5. Cox Proportional Hazards model for survival in SLS II (N=90) including additional covariates[a]

	Hazard ratio	95% CI	P-value
Age	1.05	(0.99, 1.12)	0.088
Male	1.47	(0.45, 4.80)	0.523
African American	0.55	(0.10, 3.03)	0.490
Diffuse	3.52	(0.72, 17.11)	0.119
mRSS	0.97	(0.88, 1.06)	0.456
Baseline DLCO%-predicted	0.99	(0.94, 1.03)	0.537
Baseline FVC%-predicted	1.00	(0.94, 1.07)	0.963
ΔQILD ≥2%	4.23	(1.37, 12.98)	0.012

[a] C-index for model: 0.758

e-Table 6. Cox Proportional Hazards model for survival in SLS I (N=82) using the two definitions of progression of ILD (physiologic and radiograph)[a]

	Hazard ratio	95% CI	P-value
Age	1.04	(1.00, 1.07)	0.042
mRSS	1.04	(1.00, 1.07)	0.022
Progression on PFTs[b]	1.32	(0.53, 3.29)	0.555
ΔQILD ≥2%	2.28	(1.05, 4.96)	0.037

[a] C-index for model: 0.714
[b] Defined as FVC decline ≥10% OR FVC decline between 5-9% AND DLCO decline ≥15% at 12 months.

Online supplements are not copyedited prior to posting and the author(s) take full responsibility for the accuracy of all data.

e-Table 7. Cox Proportional Hazards model for survival in SLS I (N=82) including change in FVC% predicted as a covariate[a]

	Hazard ratio	95% CI	P-value
Age	1.05	(1.01, 1.09)	0.016
Male sex	1.18	(0.51, 2.73)	0.700
African American race	1.81	(0.47, 7.00)	0.390
Diffuse subtype	1.01	(0.31, 3.21)	0.999
mRSS	1.03	(0.99, 1.09)	0.161
Baseline DLCO%-predicted	1.01	(0.98, 1.05)	0.458
Baseline FVC%-predicted	0.95	(0.91, 1.00)	0.069
Δ FVC%-predicted[b]	1.00	(0.95, 1.05)	0.970
Baseline QILD score	0.99	(0.95, 1.02)	0.464
ΔQILD ≥2%	1.88	(0.77, 4.59)	0.166

[a] C-index for model: 0.737
[b] Continuous covariate

e-Table 8. Cox Proportional Hazards model for survival in SLS II (N=90) including change in FVC% predicted as a covariate[a]

	Hazard ratio	95% CI	P-value
Age	1.06	(1.00, 1.12)	0.055
Male	0.92	(0.24, 3.55)	0.909
African American	0.41	(0.07, 2.43)	0.326
Diffuse	1.68	(0.29, 9.66)	0.558
mRSS	1.01	(0.91, 1.11)	0.879
Baseline DLCO%-predicted	0.99	(0.93, 1.04)	0.584
Baseline FVC%-predicted	1.01	(0.95, 1.09)	0.674
Δ FVC%-predicted[b]	0.93	(0.86, 1.00)	0.060
Baseline QILD score	1.03	(0.97, 1.10)	0.370
ΔQILD ≥2%	3.28	(0.95, 11.35)	0.061

[a] C-index for model: 0.804
[b] Continuous covariate

Online supplements are not copyedited prior to posting and the author(s) take full responsibility for the accuracy of all data.

e-Figure 1. Computer Aided Scoring of Quantitative Interstitial Lung Disease (QILD).

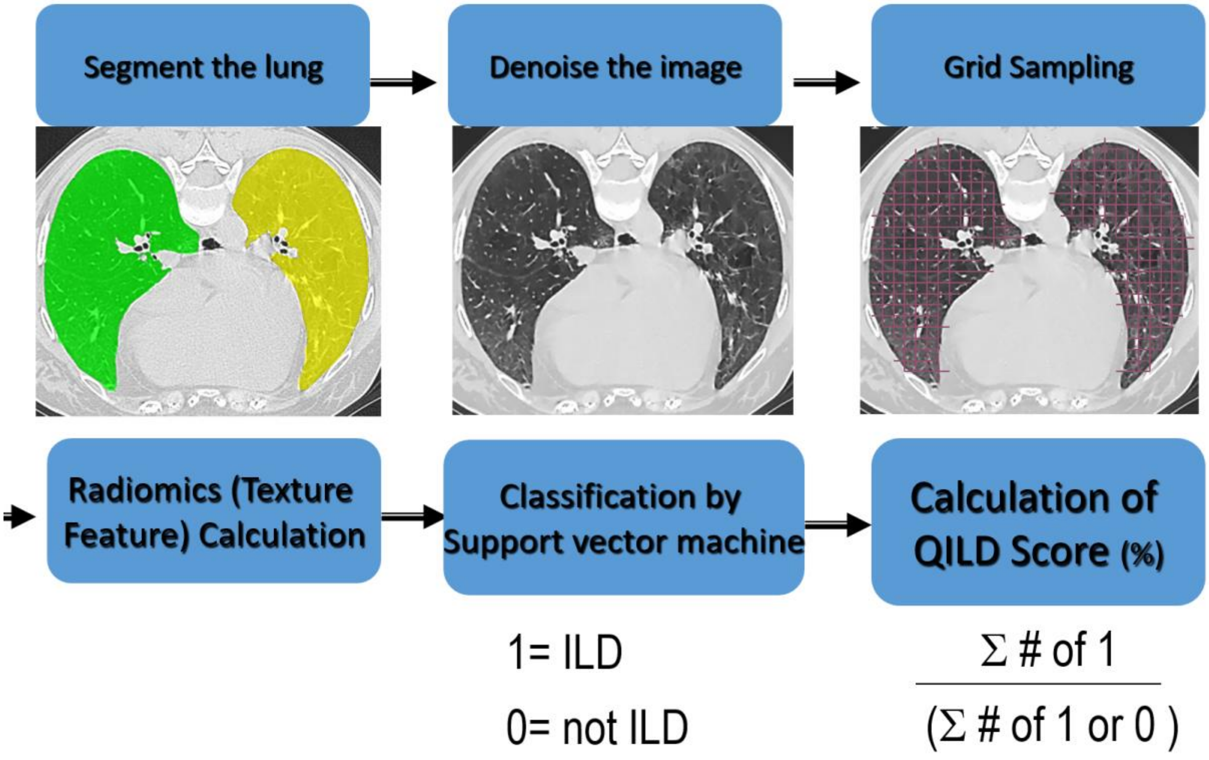

The computer system segments the lungs and lobes, denoises the images, samples a voxel from a 4-by-4 grid, performs radiomic (texture feature) based classification of voxels into quantitative ground glass (QGG), lung fibrosis (QLF) and honeycomb cysts (QHC), and calculates the quantitative interstitial lung disease (QILD) score. QILD is the sum of the three individual scores. Each score is expressed as a percentage of classified voxels/the total lung or lobe voxels (Of note that a voxel is a unit of volumetric scans and a pixel is a unit of non-volumetric scan.)

Online supplements are not copyedited prior to posting and the author(s) take full responsibility for the accuracy of all data.

e-Figure 2. Bland-Altman plot of Quantitative Interstitial Lung Disease (QILD). Technical reproducibility was estimated for the QILD score. Two technical factors were estimated: (1) random sampling within 4-by-4 grid in calculating texture features, and (2) random seed point in 5 mean clustering in quantitative ground glass (QGG) opacity. Top two clusters with lower intensities were included in QGG score. Means (±SD) were -0.066 (±0.56) for the most severe zone and -0.020 (± 0.30) in the whole lung. The limited agreements from the Bland-Altman plot were 1.12% for the most severe zone and 0.60% in the whole lung. Lin's concordance correlation coefficient (CCC) was 0.987 with 95% CI of [0.981, 0.993] in the whole lung. Pearson correlation of two QILD scores were 0.987 and 0.988, in the most severe zone and in whole lung, respectively. After this study, we set two technical parameters in order to 100% technical repeatability. We fixed the grid location in the 2nd row and 3rd column in texture feature calculation and set an initial seed point for the clustering method.

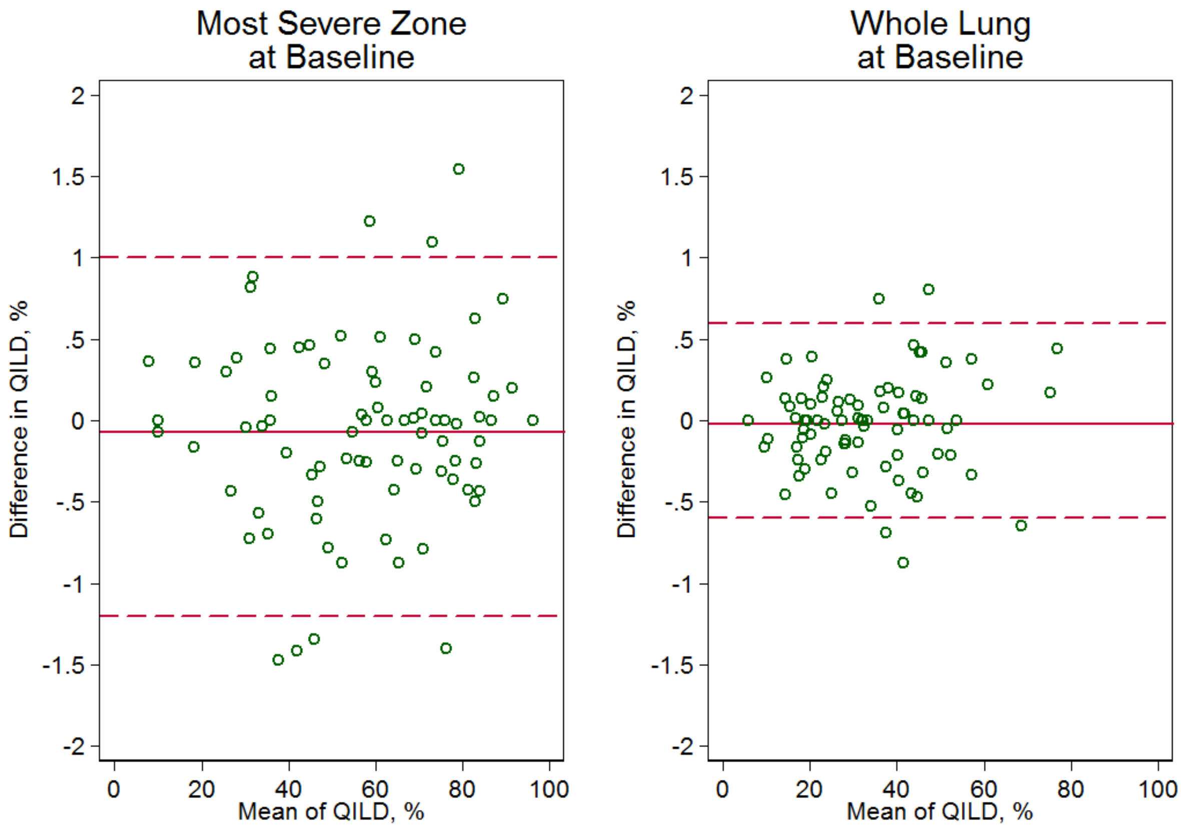

Online supplements are not copyedited prior to posting and the author(s) take full responsibility for the accuracy of all data.
